# Proceedings of the Buffalo Medical Association

**Published:** 1878-04

**Authors:** E. N. Brush

**Affiliations:** Secretary


ART. III.—*Abstract of the Proceedings of the Buffalo Medical Association.* March 5th, 1878. Reported by E. N. Brush, M. D. Secretary.

The meeting was called to order by the President, Dr. E. R. Barnes. By motion of Dr. Lothrop the minutes of the last meeting were *adopted* without reading.

Dr. E. R. Barnes read a paper an Morbus Coxarius. (See Art. I. p. 321.)

Dr. Rochester said it was a subject out of the line of his practice. As a young man he saw in the Hospitals and Dispensaries a great number of cases. A large proportion of these were children, and the cause was then looked upon as strumous. The treatment was rest, and the employment of alteratives, which were used as much as tonics and cod-liver oil are now employed. Absolute rest was insisted upon, a splint was used which insured immobility and anchylosis was the result in many cases. The treatment, being based upon a different idea as to cause, was of course varied from that now employed, when the traumatic origin of the disease was so widely held.

A few remarks were indulged in by other members present complimentary to the paper.

Dr. Rochester, under the head of voluntary communications, reported the following case. On the fifteenth of Feb. he was called in consultation with Dr. Earl of Black Rock, to see a young man aged twenty-one residing on Clinton Ave. The history was that the patient had been taken very ill on the evening of the tenth. He had a chill, followed by pain in the bowels, elevation of temperature, a rapid pulse and eventually, what was supposed to be peritonitis. When he saw the patient he had a tumid abdomen, tenderness over the right iliac region, pulse 120 and temperature 102.5°.

After a careful examination there was no difficulty in determining a circumscribed swelling which was easily isolated. He expressed the opinion that there was a perityphlitic abscess, which diagnosis Dr. Earl had already made. The patient had, four or five years previously, had an inflammation of the bowels, and two members of the *family* had died with the same disease. A cathartic had been administered which had operated, producing considerable pain and distress. Dr. Rochester suggested that the only thing which could be done was to make an operation, and fully and carefully canvassed the question with the parents. Owing to their sad experience with other members of the family the parents found it difficult to decide on the matter and held their decision for a short time. Opium and warm applications were continued.

On the 16th an operation was decided upon and was made on the afternoon of that date by Prof. J. F. Miner. There were present, Drs. Rochester, Earl, Benj. Lothrop, W. W. Miner and Brush and Mr. Bidaman, a medical student.

Chloroform was administered and a careful examination again made, resulting in confirming the diagnosis.

The swelling extended from Poupart's ligament, along the course of the colon, but a little to the right. Dr. Miner made a very careful exploratory operation, the incision being that which would be employed in ligation of the external iliac artery. When the tumor was reached it was found to be circumscribed by inflammatory adhesion. A narrow tenotomy knife was introduced and the opening subsequently enlarged, evacuating a large amount of very foul smelling pus. The cavity was thoroughly washed out by carbolized water, a drainage tube introduced and the wound closed. Previous to the operation the patient's pulse was 104, after recovering from the anæsthesia it was 84. The patient was reported on March 5th to be steadily convalescing.

Dr. Rochester, in speaking of this case, said, it was the first operation of the kind made in Buffalo of which he had any knowledge. He had seen a large number of similar cases, and had seen them terminate in various ways. One case which he saw recovered after the abscess had spontaneously opened through the abdominal walls; another had penetrated the colon, the patient had recovered. Some three or four years ago he had taken Prof. Miner to operate on a case, but found on arriving at the patient's house that the abscess had disappeared. The patient informed him that it had emptied into the bladder and proved the assertion by passing through the urethra pus and fæces. This patient still survives, but in a most pitiable condition, having a permanent opening from the bowel into the bladder.

Under the head of prevailing diseases, some erysipelas was reported, and Dr. Hauenstein mentioned some cases of puerperal fever. Influenza was also reported, some cases being of great severity.

Dr. Fowler was elected Secretary *pro tem*, for the ensuing month owing to the contemplated absence of the Secretary from the city.

